# LAMA3 DNA methylation and transcriptome changes associated with chemotherapy resistance in ovarian cancer

**DOI:** 10.1186/s13048-021-00807-y

**Published:** 2021-05-15

**Authors:** Li-yuan Feng, Yong-zhi Huang, Wei Zhang, Li Li

**Affiliations:** Department of Gynecologic Oncology, Guangxi Medical University Cancer Hospital, Key Laboratory of Early Prevention and Treatment for Regional High Frequency Tumor, Ministry of Education, Nanning, Guangxi China

**Keywords:** Ovarian cancer, Chemoresistance, LAMA3 methylation, Prognosis

## Abstract

**Objective:**

LAMA3 is a widely studied methylated gene in multiple tumors, but the relationship between chemotherapy resistance in ovarian cancer is unclear. In this study, LAMA3 methylation was predicted by bioinformatics, and the ability of LAMA3 methylation to predict the chemotherapy resistance and prognosis of ovarian cancer was confirmed in experiments.

**Methods:**

Multiple databases have performed the bioinformatics analysis of methylation and transcription factor binding site (TFBS) on the promoter region of LAMA3 gene. Pyrosequencing detected the methylation of LAMA3. QRT-PCR and immunohistochemistry detected the expression of LAMA3. Real Time Cell Analyzer (RTCA) detects changes in cell proliferation, migration and invasion ability. Flow cytometry was used to detect apoptosis.

**Results:**

CPG islands of 176 bp, 134 bp, 125 bp and 531 bp were predicted in the promoter region of LAMA3 gene. The 4 prediction results are basically overlapped. 7 transcription factor binding sites were predicted, and the one with the highest score was on the predicted CpG island located in the proximal promoter region. LAMA3 hypermethylation and low expression are both associated with chemotherapy resistance and poor prognosis in ovarian cancer. LAMA3 methylation was negatively correlated with expression. After upregulation of LAMA3, the proliferation ability of chemoresistant ovarian cancer cell decreased, while the ability of apoptosis, invasion and migration increased.

**Conclusion:**

LAMA3 hypermethylation is associated with chemotherapy resistance and poor prognosis. As a typical CpG island gene, LAMA3(cg20937934) and LAMA3(cg13270625) hypermethylation is negatively correlated with low expression. LAMA3 promotes the invasion, migration and apoptosis of SKOV3DDP. In the future, the mechanism of LAMA3 methylation in ovarian cancer will need to be further studied.

**Supplementary Information:**

The online version contains supplementary material available at 10.1186/s13048-021-00807-y.

## Introduction

Ovarian cancer is one of the three most common malignant tumors of the female reproductive system, and the mortality rate is the highest in gynecological malignancies [1]. Because of a lack of early indicators and symptoms, 70% of patients are diagnosed at an advanced stage [2, 3]. The standard treatment for ovarian cancer patients at the first diagnosis is optimal debulking combined with platinum-based chemotherapy, but only 70–80% of patients are effective in combination chemotherapy, and most women undergoing treatment will eventually develop chemotherapy resistance [4]. Chemotherapy resistance greatly reduces the survival rate of ovarian cancer patients, which is an important reason for the high mortality [5]. Therefore, there is an urgent need for early markers of chemotherapy sensitivity and therapeutic targets to reverse drug resistance [6].

Chemotherapy resistance in ovarian cancer is related to many factors such as genetics, epigenetics, somatic gene mutations, immune system and tumor microenvironment abnormalities [7, 8]. DNA methylation is the most widely studied subject of cancer epigenetics [9]. The methylation status of CpG island in gene regulatory region is closely related to gene expression. Abnormal DNA methylation alters gene expression and affects gene function by interfering with the binding of specific transcription factors to the recognition positions in gene promoters [10, 11]. Chemotherapy drugs kill tumor cells by causing DNA damage, inhibiting proliferation, and promoting cell apoptosis. Abnormal methylation levels of the promoters of these related pathway genes can affect the sensitivity of tumor cells to chemotherapy drugs.

Current studies have reported that at least hMLH1, BRCA1, FANCF, RASSF1A, Hsulf-1, PARP1, CDKN1A, PD-CD4, WWOX, DOK2, SOCS2, FBX032, HOXA10 are abnormal methylated genes associated with chemoresistance of ovarian cancer [12–15]. As a marker of chemotherapy resistance in ovarian cancer, DNA methylation has advantages such as chemical stability, quantitative analysis, chemoresistance related methylation changes usually occur before the start of chemoresistance, and non-invasive detection (which can be detected in the patient’s body fluids) [16, 17]. At present, there is no reliable method to predict chemoresistance of ovarian cancer. DNA methylation is a potential candidate molecular marker.

Compared with gene mutation, epigenetics can be reversed. Therefore, demethylation drugs are expected to become a new generation of targeted drugs for cancer treatment [18]. Although no demethylation drug has been approved for the clinical treatment of ovarian cancer patients, several clinical trials have shown that demethylation drug (decitabine) can improve the chemotherapy sensitivity and prolong the prognosis of patients with platinum-resistant ovarian cancer patients [19–21]. For example, Fang [22] used carboplatin combined with SGI-110 (second-generation demethylation drug) or standard chemotherapy to treat patients with relapsed platinum-resistant ovarian cancer. It was found that the 6-month PFS median survival rate of carboplatin combined with SGI-110 treatment group was 37%, and that of standard chemotherapy group was 13%. It shows that SGI-110 treatment induces platinum resensitivity and has clinical activity.

LAMA3 (Laminin subunit Alpha 3) is a widely studied methylated gene in various tumors. LAMA3 abnormal methylation is involved in the occurrence, development and prognosis of various malignant tumors such as pancreatic cancer, gastric cancer, head and neck tumors, and lung cancer [23–26]. LAMA3 is important in development by interacting with other extracellular matrix components. LAMA3 and its variants expressions are exacerbated that increase the motility and invasiveness in ovarian cancer [27, 28]. Tang found that LAMA3 is abnormally hypermethylated in ovarian cancer, but the relationship between LAMA3 methylation and chemotherapy resistance in ovarian cancer has not been reported. In this study, we used bioinformatics to predict LAMA3 methylation and confirmed its ability to predict chemotherapy resistance and prognosis of ovarian cancer.

## Materials and methods

### CpG island prediction in the promoter region of LAMA3 gene

The 5 KB upstream region sequence (promoter region) of the human LAMA3 gene was obtained from NCBI database. The prediction of CpG island was carried out by using the database of Methprimer, CpG Finder, EMBOSS and MethCancer. The conditions were set as follows: Observed/Expected ratio > 0.60, Percent C + Percent G > 50.00, Length > 100.00.

### Transcription factor binding sites prediction in the promoter region of LAMA3 gene

The 5 KB upstream region sequence (promoter region) of LAMA3 gene was selected to study the possible relationship between CpG island methylation and transcription factor binding. FPROM, CNNPromoter program and Promoter 2.0 Prediction Server database were used to search for transcription factor binding sites in CpG island of LAMA3 gene promoter.

### Protein-protein interaction and pathway/biological process enrichment

Protein-Protein Interaction analysis (PPI) for LAMA3 was performed by STRING database (https://string-db.org/). Download KEGG and GO biological process data under the analysis module of the String website. The histogram was drawn with observed gene count as the abscissa and term description as the ordinate. KEGG pathway and GO biological process enrichment analysis were conducted using LAMA3 interactive genes.

### Patient samples

From March 1997 to April 2013, epithelial ovarian cancer patients with chemotherapy outcome were collected from the Guangxi Medical University Cancer Hospital. 188 paraffin tissues were used for immunohistochemistry, and 71 frozen tissues were used for pyrosequencing and QRT-PCR. All patients had chemotherapy outcomes and postoperative pathology. All Samples received the patient’s informed consent and the approval of the Ethics Committee of the Guangxi Medical University Cancer Hospital. Chemoresistance was defined as the patients who do not achieve a complete response after initial treatment, or relapse within 6 months after complete response. Chemosensitivity was defined as the patients who recurrence more than 6 months after complete response. Overall survival (OS) was defined as the time from diagnosis to death due to ovarian cancer. Progression-free survival (PFS) was defined as the time from initial treatment to tumor progression.

### Pyrosequencing

DNA extraction and Bisulfite conversion were performed in accordance with the instructions of GeneJET Genomic DNA Purification Kit (Thermo Scientific, Cat. No.K0721) and EpiTect Bisulfite Kit (48) (QIAGEN, Cat. No.59104). Perform pyrosequencing on PYROMARK Q96 ID (QIAGEN) according to the instructions of PyroMark® PCR Kit (QIAGEN, Cat. No.978705). According to the previous 450 K Infinium Methylation BeadChip in our laboratory, the detection sites were LAMA3(cg20937934) and LAMA3(cg13270625) in the TSS 200 region of LAMA3. See Supplementary File 1 for primer sequences.

### RNA extraction and QRT-PCR

RNA extraction and reverse transcription were performed according to the Genomic RNA Purification Kit (Thermo Scientific, Cat. No. K0731) and Revert Aid First Strand cDNA Synthesis Kit (Thermo Scientific, Cat. No. K1622) instructions. One Step TB Green® PrimeScriptTM RT-PCR Kit (Takara, Cat. No. RR066B) was used for real-time PCR on an ABI step-one plus PCR machine. See Supplementary File 2 for primer sequences.

### Tissue microarray and immunohistochemistry

The pathological types were confirmed by HE stain. Each tumor has 2–3 repeated tissue spots. The diameter is 1 mm. Immunohistochemistry was performed according to the ready-to-use immunohistochemical ultrasensitive UltraSensitiveTM SP test kit (maixin, Cat. No. KIT-9710) instructions. LAMA3 concentration is 1:200 (Abcam, Cat. No.ab242197). Two pathologists read the pathological sections independently. The score criteria are as follows: Positive cell ratios of < 1, 1–25%, 25–50%, 50–75% and 75–100% were 0, 1, 2, 3, 4 points, respectively. Stain intensity of no coloring, light yellow, yellow, brown were 0, 1, 2, 3 points, respectively. The product of positive cell ratio and stain intensity is stain index. Stain index≤6 points was classified as low expression, while > 6 points was classified as high expression [29].

### Cell lines and transfection

Human ovarian cancer cell line SKOV3 and cisplatin resistant cell line SKOV3DDP came from the Key Laboratory of Early Prevention and Treatment for Regional High Frequency Tumor, Ministry of Education, Guangxi Medical University (drug resistance index 2.64). The cells were cultured in DMEM medium (Corning, Cat. No. D6429) containing 10% FBS, 1% penicillin and streptomycin. LAMA3 stable overexpression of SKOV3DDP was performed using SAM two-vector lentivirus. Stable transfected cells were screened with 1μg/ml puromycin for 72 h. The cell lines overexpressing LAMA3 (SKOV3DDP-LAMA3-High) and blank control (SKOV3DDP-LAMA3-NC) were successfully constructed.

### In vitro functional experiment

Real time cell analysis (RTCA) was used to observe the cell growth. The cells were inoculated at the appropriate cell density and cultured for 120 h to obtain the real-time dynamic curve of cell growth, migration and infiltration. According to the IC50 value of the cells, two cisplatin concentrations are set: IC50 and 1.25 × IC50. After 24 h of cisplatin treatment, apoptosis rate was detected by flow cytometry.

### Statistical analysis

SPSS17.0 was used for analysis. T test (measurement data) and chi-square test (categorical data) were used for comparison between the two groups. ROC analysis confirmed the ability of LAMA3 to predict chemotherapy outcomes. The association between LAMA3 expression and prognosis was assessed using Kaplan-Meier plotter. The correlation between LAMA3 methylation and expression was assessed with Spearman test. *P* values were two-sided, and *P* < 0.05 was considered statistically significant.

## Results

### CpG island prediction in the promoter region of LAMA3 gene

**Fig. 1:**
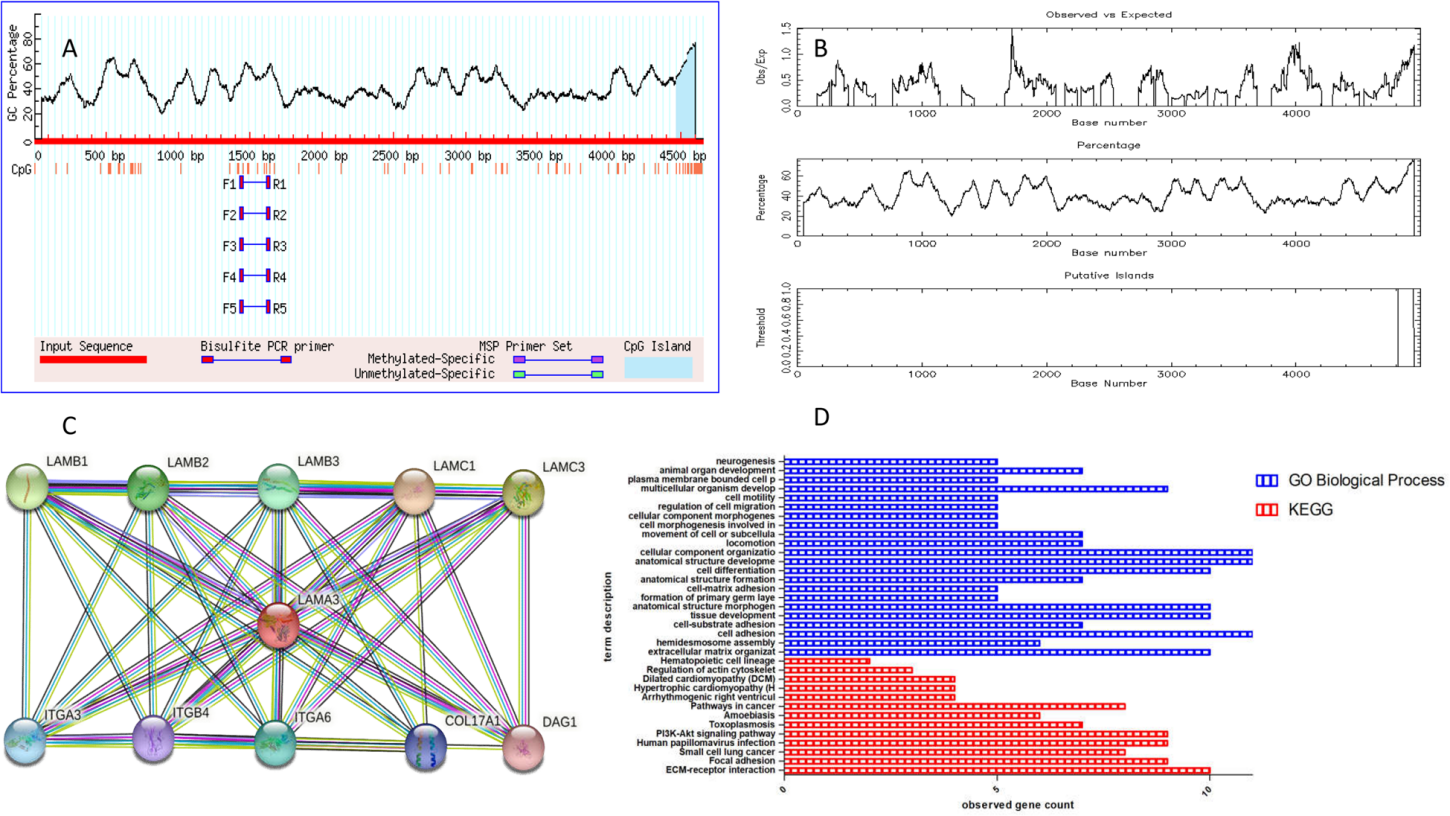
**a**. Prediction of CpG island in the promoter region of lama3 gene (MethPrimer). **b**. Prediction of CpG island in the promoter region of lama3 gene (EMBOSS). **c**. Lama3 interaction genes (PPI). **d**. KEGG/GO biological process enrichment of Lama3 interaction genes

Using MethPrimer, CpG Finder, EMBOSS and MethCancer respectively predicted a 176 bp (see Fig. 1a), 134 bp, 125 bp (see Fig. 1b), 531 bp CPG island in the promoter region of the LAMA3 gene. The 4 prediction results basically overlap. It can be seen that the CG distribution is very dense in the 500 bp upstream region containing the transcription start site (TSS). The proximal promoter region is generally considered to be the main regulatory domain. Therefore, the LAMA3 gene is a typical CpG island gene. LAMA3 transcription factor binding and gene expression may be affected by methylation.

### Transcription factor binding sites prediction in the promoter region of LAMA3 gene

The 5 KB upstream region sequence (promoter region) of LAMA3 gene was obtained. FPROM results showed that 2 transcription factor binding sites were predicted in the upstream promoter region of the LAMA3 gene. The one with the highest score was the predicted CpG island located in the proximal promoter region (position:4418,LDF:+ 2.926;position:795,LDF:-0.874;). CNNPromoter results showed that there are 7 transcription factor binding sites in the upstream promoter region of the LAMA3 gene, and the one with the highest score is also the predicted CpG island located in the proximal promoter region (The top four scores are: position:4700, SCORE:1.123; position:3000,SCORE:0.71; position:2300,SCORE:0.686; position:1600,SCORE:0.635; position:900,SCORE:0.669;). Promoter 2.0 Prediction Server results showed that there are 5 transcription factor binding sites in the upstream promoter region of the LAMA3 gene, and the one with the highest score is also the predicted CpG island located in the proximal promoter region (position:4700, SCORE:1.123; position:3000,SCORE:0.71; position:2300,SCORE:0.686; position:1600,SCORE:0.635; position:900,SCORE:0.669;). Transcription factor binding sites were all predicted to be highly overlapping with CpG Island in LAMA3 proximal promoter region. This indicates that LAMA3 CpG methylation is closely related to transcription factor regulation. It is also suggested that epigenetics may play an important role in LAMA3 gene expression.

### PPI and KEGG/GO biological process enrichment

PPI analysis using STRING revealed that 10 genes including LAMB1,LAMB2, LAMB3, LAMC1, LAMC3, ITGA3, ITGA4, ITGA6, COL17A1 and DAG1 were interacted with LAMA3 (Fig. 1c). KEGG pathway enrichment of LAMA3 interactive genes showed that ECM-receptor interaction, focal adhesion, PI3K-Akt signaling pathway, human papillomavirus infection were the most enriched pathways (Fig. 1d). Additionally, extracellular matrix organization, cell adhesion, tissue development, cell differentiation, cell migration, neurogenesis were most enriched GO biological process of LAMA3 interactive genes (Fig. 1d).

### Characteristics of patients

188 patients met the eligibility criteria for inclusion. The clinical characteristics of the patients are shown in Table 1.The mean age of patients was 51.87 ± 11.45 years. According to FIGO standard staging, there were 133 cases in stage III-IV and 55 cases in stage I-II. Histological types: 125 cases of serous carcinoma, 13 cases of mucinous carcinoma,50 cases of other epithelial ovarian cancer. Cell differentiation: 14 patients with high differentiation, 18 patients with medium differentiation and 156 patients with low differentiation. There were 124 patients with chemotherapy sensitivity and 64 patients with chemotherapy resistance.

**Table 1:** Clinical characteristics of patients

Clinical characteristics	All(188)
Age	51.87 ± 11.45
FIGO stage	
I-II	55 (29.26%)
III-IV	133 (70.74%)
Grade	
3	156 (82.98%)
2	18 (9.57%)
1	14 (7.45%)
Missing	0
Histology types	
Serous	125 (66.49%)
Mucinous	13 (6.91%)
Others	50 (26.60%)
surgical debulking	
Optimal	148 (78.72%)
Suboptimal	40 (21.28%)
Missing	0
Chemotherapy outcome	
chemosensitive	124 (65.96%)
chemoresistant	64 (34.04%)

### LAMA3 hypermethylation is associated with chemotherapy resistance and poor prognosis

**Fig. 2:**
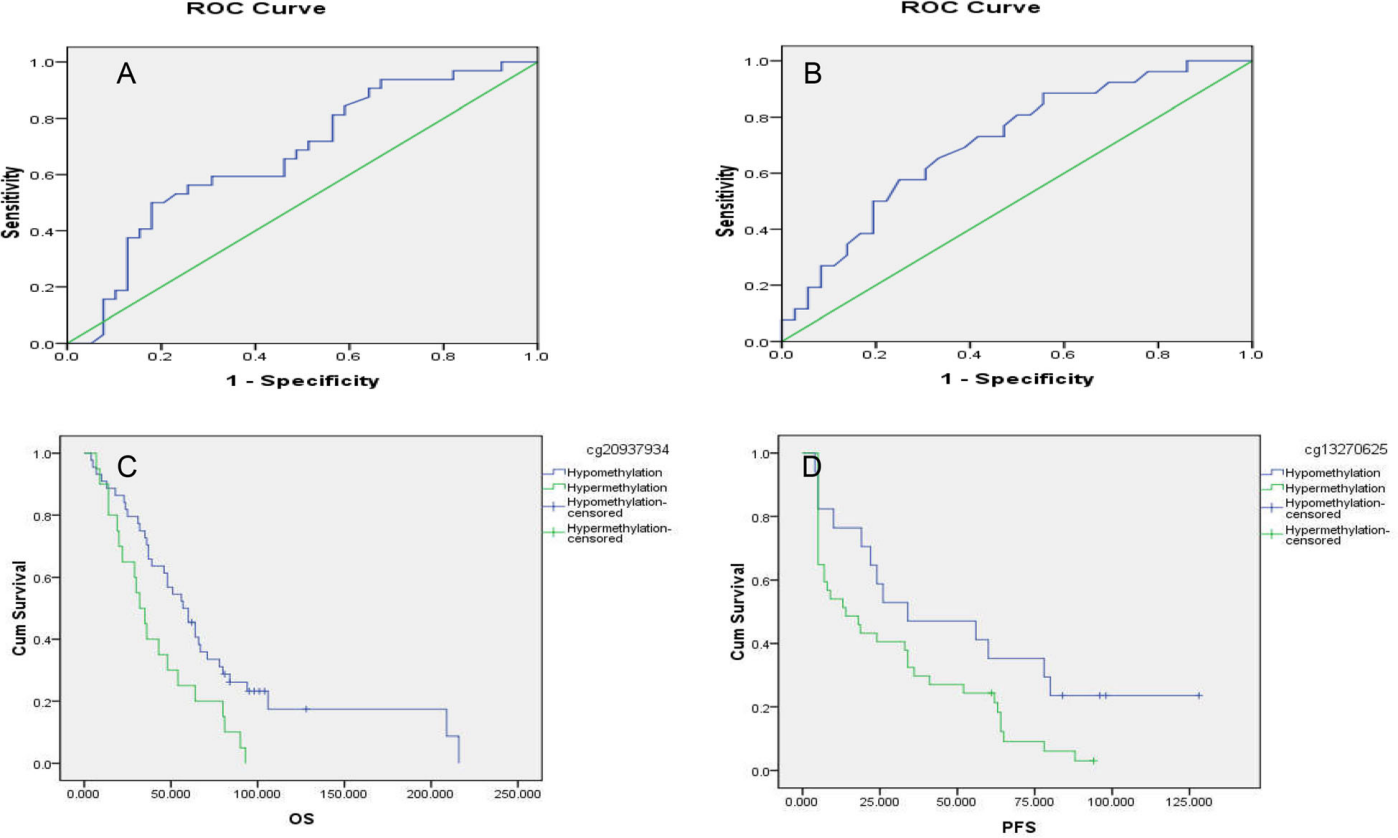
**a**. The ROC curve of LAMA3 (cg20937934) in predicting chemotherapy resistance. **b**. The ROC curve of LAMA3 (cg13270625) in predicting chemotherapy resistance. **c**. Association of LAMA3(cg20937934) methylation with OS. **d**. Association of LAMA3 (cg13270625) methylation with PFS

In order to investigate the accuracy of bioinformatics prediction of CpG island methylation in the promoter region of LAMA3 gene, this study selected two locis (cg20937934 and cg13270625) in the LAMA3 TSS200 region for methylation detection. The results showed that the methylation level of LAMA3 (cg20937934) and LAMA3 (cg13270625) in chemoresistant ovarian cancer patients was significantly higher than that in chemosensitive ovarian cancer patients (cg20937934:0.72 ± 0.16 VS 0.61 ± 0.21, *P* = 0.01; cg13270625:0.57 ± 0.13 VS 0.44 ± 0.18, *P* = 0.002). ROC analysis showed that the AUC of LAMA3 (cg20937934) and LAMA3 (cg13270625) to predict chemotherapy sensitivity were 0.67(95%CI 0.542–0.796, *P* = 0.015) and 0.71(95%CI 0.582–0.839, *P* = 0.005) respectively. The ROC curve was shown in Fig. 2a and b. Kaplan-meier survival analysis showed that OS was shorter in patients with hypermethylation of LAMA3 (cg20937934), but the differences were not statistically significant for PFS. PFS were shorter in patients with hypermethylation of LAMA3(cg13270625), but the differences were not statistically significant with OS. See Fig. 2c and d.

### Low expression of LAMA3 is associated with chemotherapy resistance and poor prognosis

**Fig. 3:**
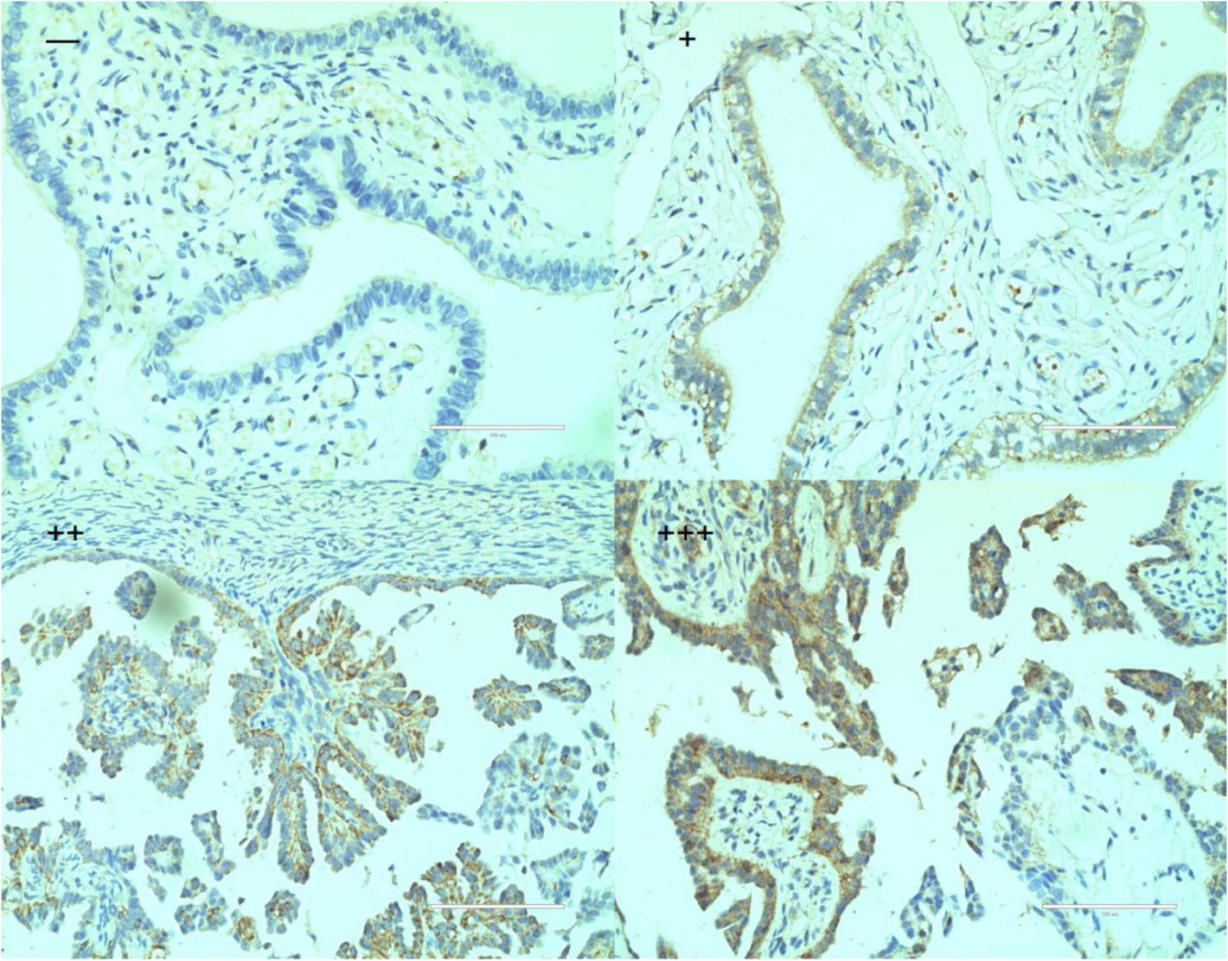
Immunohistochemical stain (400×)

**Fig. 4:**
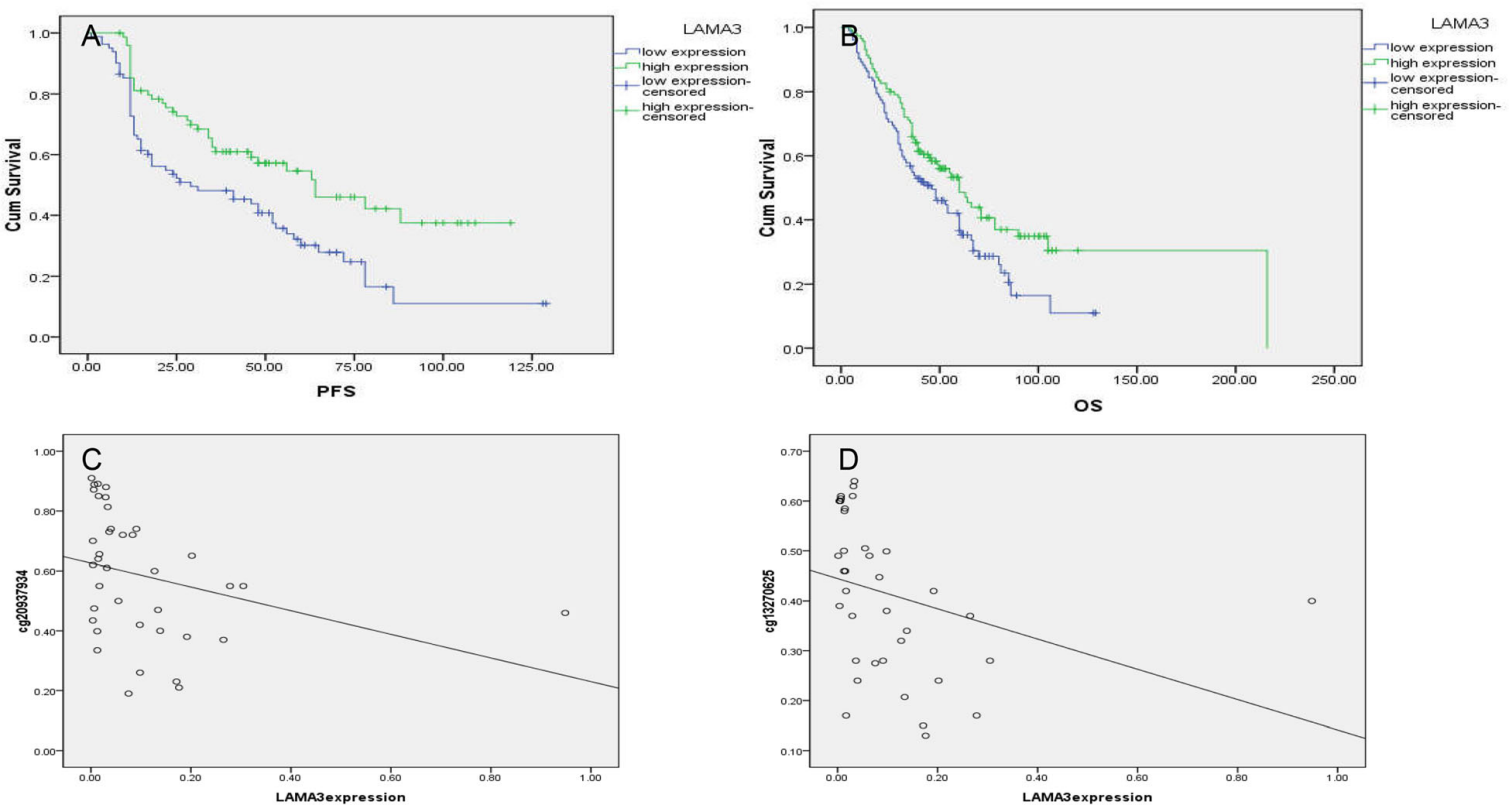
**a**. Association of LAMA3 expression with PFS. **b**. Association of LAMA3 expression with OS. **c**. Association of LAMA3(cg20937934) methylation with LAMA3 mRNA expression. **d**. Association of LAMA3(cg13270625) methylation with LAMA3 mRNA expression

QRT-PCR results showed that compared with chemotherapy sensitive patients, the expression of LAMA3 was down-regulated 2.58 times in chemotherapy resistant patients (*P* = 0.025). Immunohistochemistry showed that LAMA3 protein was highly expressed in 67/124 (54.03%) chemotherapy sensitive patients and 25/64 (39.06%) chemotherapy resistant patients. Compared with chemotherapy sensitive patients, the expression of LAMA3 protein in chemotherapy resistant patients was significantly reduced (*P* = 0.047). The AUC of LAMA3 protein expression predicting chemotherapy resistance in ovarian cancer was 0.58 (95% CI 0.489–0.661, *P* = 0.093). LAMA3 is mainly expressed in the cytoplasm, and a small amount is expressed in the cell membrane, see Fig. 3. Ovarian cancer patients with low LAMA3 expression are associated with poorer OS and PFS (OS:60.00(95%CI 46.63–73.57) VS 46.00(95%CI 31.51–60.49),*P* = 0.021; PFS:64.00(95%CI 36.71–91.29) VS 29.00(95%CI 3.97–54.03), *P* = 0.002). The survival curve is shown in Fig. 4a and b.

### LAMA3 hypermethylation is negatively correlated with low expression

In order to investigate whether CpG island methylation in LAMA3 gene promoter affects transcription factor binding regulatory expression, we analyzed the correlation between CpG island methylation in LAMA3 promoter region and LAMA3 mRNA expression level. The spearman correlation analysis results showed that the methylation levels of LAMA3 were negatively correlated with the expression of LAMA3 mRNA (cg20937934:R = -0.455, *P* = 0.004; cg13270625:R = -0.624, *P* = 0.000). The scatter plot is shown in Fig. 4c and d.

### LAMA3 promotes the invasion and migration of SKOV3DDP

**Fig. 5:**
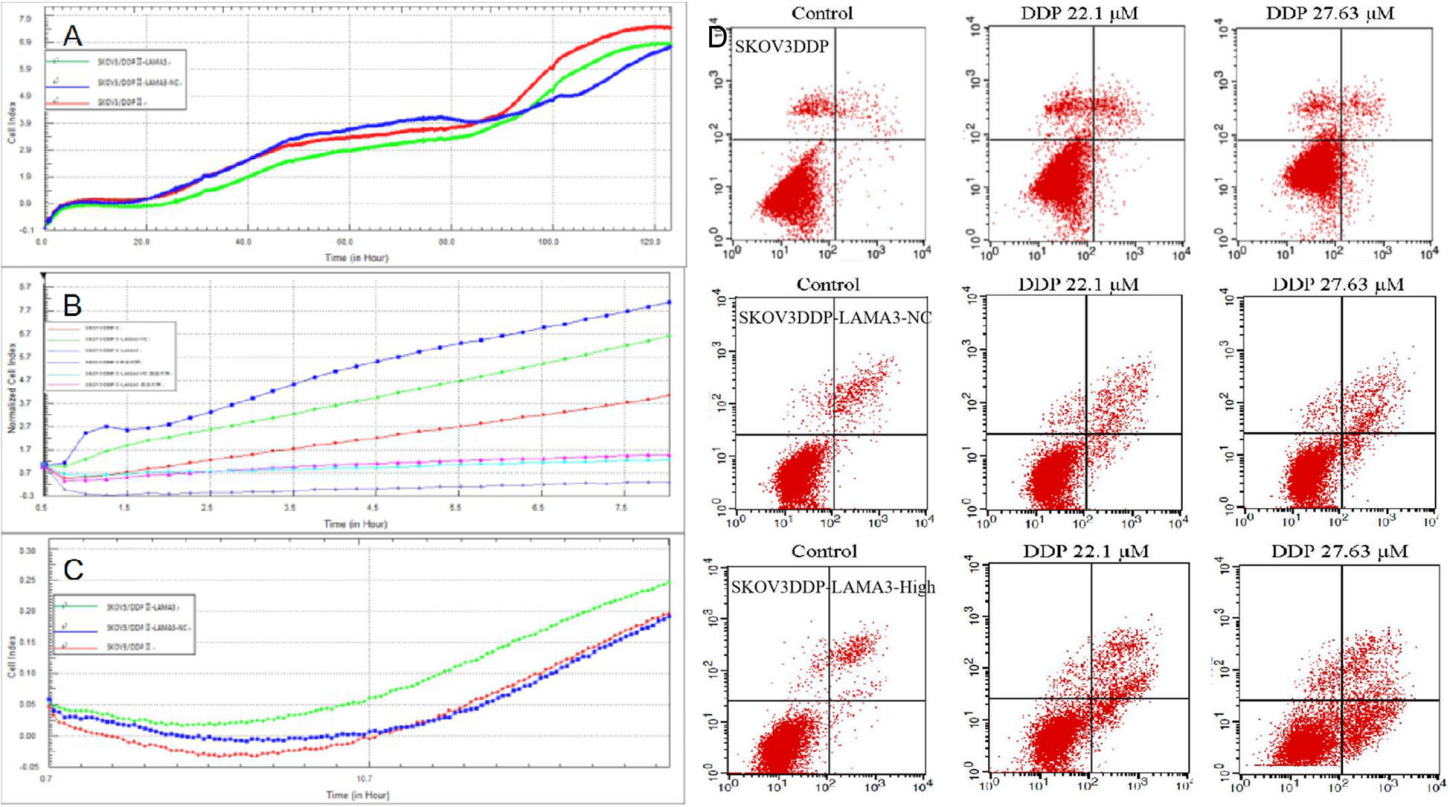
**a**. Growth curves of SKOV3DDP, SKOV3DDP-LAMA3-NC and SKOV3DDP-LAMA3-High cells. **b**. Migration Experiment (RTCA). **c**. Invasion experiment (RTCA). **d**. Apoptosis rate of SKOV3DDP, SKOV3DDP-LAMA3-NC and SKOV3DDP-LAMA3-High cells

RTCA results showed that SKOV3DDP, SkOV3DDP-LAMA3-NC and SkOV3DDP-LAMA3-high cells grew slowly and their growth rates were basically the same when cultured for 0–45 h. Starting from 80 h, the growth rate of the three types of cells was significantly accelerated, and the growth rate of SkOV3DDP-LAMA3-high was significantly lower than that of SKOV3DDP and SKOV3DDP-LAMA3-NC cells. See Fig. 5a. Invasion and migration experiments showed that the CI value of SKOV3DDP-LAMA3-High was significantly higher than that of SKOV3DDP, indicating that the invasion and migration ability of chemotherapy resistance ovarian cancer cells was significantly enhanced after overexpression of LAMA3. See Fig. 5b-c.

### LAMA3 promotes the apoptosis of SKOV3DDP

Recent studies have confirmed that the dysfunction of apoptosis is a key factor in the development of chemotherapy resistance. We tested whether the LAMA3 gene is involved in the process of apoptosis induced by chemotherapy drugs. Flow cytometry results showed that the apoptosis rates of SKOV3DDP, SKOV3DDP-LAMA3-NC and SKOV3DDP-LAMA3-High treated with 22.1umol/L cisplatin concentration for 24 h were 8.05 ± 1.21, 6.19 ± 0.10 and 13.58 ± 0.40; The apoptosis rates of SKOV3DDP, SKOV3DDP-LAMA3-NC and SKOV3DDP-LAMA3-High treated with 27.63umolL cisplatin concentration for 24 h were 9.86 ± 1.73, 8.10 ± 0.05 and 19.97 ± 0.38. Under the same conditions of cisplatin, the apoptosis rate of SKOV3DDP-LAMA3-High cells was significantly higher than that of SKOV3DDP. Apoptosis rate is shown in Fig. 5d.

## Discussion

Chemotherapy resistance is the main clinical obstacle in the treatment of ovarian cance. Previous prognostic indicators such as age, stage, and histological type are not enough to predict the efficacy of conventional chemotherapy [30]. Therefore, new biomarkers to predict chemotherapy resistance and treatment strategies to overcome resistance are needed. DNA methylation biomarkers may be useful in screening for chemoresistance in ovarian cancer, because of their stable chemical properties and the methylation changes associated with chemoresistance usually occur before chemoresistance. Several clinical trials have shown that demethylation drugs are effective in the treatment of ovarian cancer chemotherapy resistance.

LAMA3(Laminin subunit Alpha 3) is a widely studied methylated gene in many tumors. However, the molecular mechanism of LAMA3 in ovarian cancer and its relationship with chemotherapy sensitivity is not clear. In this study, we used bioinformatics to predict LAMA3 methylation and confirmed its ability to predict chemotherapy resistance and prognosis of ovarian cancer. As far as we know, this is the first study to link LAMA3 methylation with chemotherapy resistance and clinical prognosis in ovarian cancer.

In our study, the prediction results of CpG island show that LAMA3 gene is a very typical CpG island gene. The binding sites of transcription factor and LAMA3 gene promoter overlap highly with the predicted CPG island fragment, suggesting that transcription factor binding and gene expression may be regulated by DNA methylation. As a laminin coding gene, LAMA3 has been proved to be involved in the occurrence and development of tumors, promote the proliferation of tumor cells, and affect the invasion and metastasis of tumors. Our KEGG pathway and GO biological processes results indicate that LAMA3 may participate in ECM-receptor interaction, focal adhesion, PI3K-Akt signaling pathway, human papillomavirus infection, cell adhesion, cell differentiation, cell migration.

In order to investigate the accuracy of bioinformatics prediction of LAMA3 methylation, we examined the methylation and expression levels of LAMA3 in ovarian cancer patients with chemotherapy outcomes. In chemoresistance ovarian cancer patients, LAMA3 has abnormally high methylation and low expression. LAMA3 hypermethylation is negatively correlated with low expression. The AUC of LAMA3 methylation predicting chemotherapy resistance is 0.67 and 0.71, indicating that the LAMA3 methylation level has a certain diagnostic value for the chemotherapy outcome of ovarian cancer patients. Our data also showed that the PFS and OS of patients were significantly reduced in patients with LAMA3 hypermethylation and low expression, which is consistent with Tang’s conclusion. Our data further strengthen the findings that low LAMA3 expression is associated with poor prognosis in ovarian cancer, and that this low expression is likely to be regulated by methylation.

High methylation and low expression of LAMA3 are found in breast cancer, lung cancer, bladder cancer and other malignant tumors [31–33]. Abnormal LAMA3 levels affect the synthesis of laminin, hinder the formation of basement membrane [34], and promote tumor cell invasion and metastasis [35]. Our data found that overexpression of LAMA3 in SKOV3DDP increased the ability of invasion and migration. Consistent with findings in other cancers, LAMA3 promotes the invasion and migration of chemoresistant ovarian cancer cell. It is well known that apoptosis dysfunction is a key factor in the occurrence of chemoresistance [36, 37]. Most chemotherapeutic drugs cause tumor cells death through apoptosis. The deletion of pro-apoptotic genes or the overexpression of anti-apoptotic genes will lead to chemoresistance [38, 39]. We found that overexpression of LAMA3 enhanced the apoptosis induced by cisplatin in SKOV3DDP.

There are some shortcomings in this study. First, we can only analyze a small number of patients because it is difficult to extract sensitive tissues and corresponding resistant tissues of ovarian cancer. Second, the factors affecting the chemotherapy resistance of ovarian cancer are complex and diverse. Predicting chemotherapy resistance is difficult, especially for single biomarker. Although the LAMA3 methylation level predicts chemotherapy resistance in ovarian cancer has certain predictive value, it still needs to be combined with other biomarkers to further improve the predictive efficacy. Third, LAMA3 overexpression increases the apoptosis, invasion and migration of chemoresistant ovarian cancer cell. Immunohistochemistry of proteins involved in EMT transition (E-Cadherin, N-Cadherin, Vimentin) can be shown to support the data for increased migration. Further study of these proteins will be needed in the future. Its key signaling pathways need to be further studied to further clarify the mechanism of LAMA3 involvement in chemotherapy resistance.

## Conclusions

In summary, our study demonstrates that LAMA3 hypermethylation is associated with chemotherapy resistance and poor prognosis. As a typical CpG island gene, LAMA3(cg20937934) and LAMA3(cg13270625) hypermethylation is negatively correlated with low expression. LAMA3 promotes the invasion, migration and apoptosis of SKOV3DDP. In the future, the mechanism of LAMA3 methylation in ovarian cancer will need to be further studied.

## Supplementary Information

**Additional file 1: Table S1.** Sequencing primer. **Table S2.** The primer sequences of QRT-PCR.

## Data Availability

Not applicable.

## Abbreviations

LAMA3: Laminin subunit Alpha 3
TFBS: Transcription factor binding site
RTCA: Real Time Cell Analyzer
PPI: Protein-Protein Interaction analysis
TSS: Transcription start site
ROC: Receiver Operating Characteristic
AUC: Area Under Curve
OS: Overall survival
PFS: Progression-free survival
FIGO: The international federation of gynecology and obstetrics
